# Evidence-Based Management of Heart Failure: Clinical Impact and National Health Service Implications

**DOI:** 10.7759/cureus.92735

**Published:** 2025-09-19

**Authors:** Rudransh Guleria, Amandeep Singh, Hardil P Majmudar, Zi Yi Ang, Prashant Awasthi

**Affiliations:** 1 General Surgery, North Tees and Hartlepool NHS Foundation Trust, Stockton-on-Tees, GBR; 2 Internal Medicine, University Hospital Coventry and Warwickshire, Coventry, GBR; 3 General Surgery, ND Desai Medical College and Hospital, Nadiad, IND; 4 General Surgery, University Hospital of North Tees, Stockton-on-Tees, GBR; 5 Trauma and Orthopaedics, Royal Free Hospital, London, GBR

**Keywords:** effective part of multidisciplinary team, guideline directed medical therapy, heart failure, nhs hospitals, public health program development

## Abstract

Heart failure is a leading cause of morbidity and mortality worldwide and poses a major health challenge in the United Kingdom (UK), where it affects a substantial proportion of the population and consumes a substantial proportion of National Health Service (NHS) resources. Despite therapeutic advances, prognosis remains poor, and recurrent hospital admissions contribute significantly to the overall burden.

This review summarises current strategies for heart failure management, including pharmacological therapy, device-based interventions, lifestyle modification, and multidisciplinary team approaches. Particular emphasis is placed on the importance of early diagnosis, timely optimisation of therapy, and the integration of holistic care models to improve outcomes. Health inequalities remain a pressing concern, with deprived populations experiencing disproportionate disease burden and reduced access to specialist care. Addressing these challenges through community-based services, digital health solutions, and equitable allocation of resources is essential to reduce admissions, enhance survival, and alleviate pressure on healthcare systems.

## Introduction and background

Heart failure is a progressive clinical syndrome characterised by symptoms such as breathlessness, fatigue, and fluid retention, often accompanied by signs including pulmonary crackles or peripheral oedema. It arises from structural and/or functional cardiac abnormalities that lead to reduced cardiac output and/or elevated intracardiac pressures at rest or during stress, ultimately resulting in inadequate perfusion to meet the body’s metabolic demands. It represents the final common pathway of a wide range of cardiovascular diseases and remains a major cause of hospitalisation and death worldwide. The World Health Organization estimates that cardiovascular disease accounts for approximately 16% of global deaths, with coronary heart disease (CHD) as the largest single contributor [1].

In the United Kingdom, heart failure poses a substantial health challenge, affecting more than one million people with nearly 200,000 new diagnoses annually [2,3]. Prognosis remains poor, with almost half of patients dying within five years of diagnosis [4]. The economic impact is equally significant, with heart failure accounting for close to 2% of the NHS budget (around £2 billion annually), much of which is driven by unplanned hospital admissions [5,6]. These admissions are often prolonged, associated with functional decline, and represent a major target for health service improvement.

CHD is the leading cause of heart failure in the UK, while other important contributors include hypertension, valvular heart disease, arrhythmias, and cardiomyopathies [7]. Risk factors such as diabetes, obesity, smoking, and sedentary behaviour accelerate disease progression. Beyond biomedical risk, social determinants play a critical role: people living in the most deprived areas are almost twice as likely to die prematurely from cardiovascular disease compared with those in the least deprived, reflecting disparities in lifestyle risk factors, comorbidities, and access to care [8]. Addressing such inequalities is a priority of the NHS Long Term Plan, which advocates for earlier diagnosis, wider use of evidence-based therapies, and expansion of community-based care pathways [5].

Over the past two decades, substantial advances in the management of heart failure have transformed outcomes with landmark trials such as CONSENSUS (angiotensin-converting-enzyme (ACE) inhibitors) [8], PARADIGM-HF (angiotensin receptor-neprilysin inhibitors (ARNi)) [9], RALES (mineralocorticoid receptor antagonists (MRAs)) [10], SHIFT (ivabradine) [11], EMPEROR-Reduced (empagliflozin) [12] and DAPA-HF (sodium-glucose cotransporter 2 inhibitors (SGLT2i) [13]. EMPEROR-Reduced (empagliflozin) [12] has established strong mortality and morbidity benefits across diverse patient populations. Guideline-directed medical therapies such as ACE inhibitors or ARNis, beta-blockers, MRAs, and SGLT2 inhibitors have consistently been shown to improve survival and reduce hospitalisations. Device-based therapies, including implantable cardioverter defibrillators (ICDs) and cardiac resynchronization therapy (CRT), offer additional prognostic and symptomatic benefits in carefully selected patients [14]. Nevertheless, challenges remain in implementing these interventions effectively in routine practice, particularly in older patients, those with multiple comorbidities, or individuals from socioeconomically disadvantaged backgrounds.

This review specifically examines evidence-based management of heart failure with an emphasis on pharmacological therapies, device-based interventions, lifestyle modification, and multidisciplinary care within the NHS framework. Special attention is given to health inequalities, which remain a major barrier to optimal outcomes.

## Review

Methods

This article is a narrative review of current strategies for the management of heart failure, with a particular focus on clinical practice and health service implications in the United Kingdom. Literature searches were conducted in PubMed and Google Scholar using combinations of keywords including “heart failure,” “management,” “guideline-directed medical therapy,” “cardiac resynchronisation therapy,” “SGLT2 inhibitors,” “health inequalities,” and “National Health Service.”

Epidemiology and disease burden

Heart failure represents a significant and growing health challenge in the United Kingdom. The prevalence continues to rise, largely due to an ageing population and improved survival following acute coronary events [2-4]. NHS data indicate that heart failure care consumes approximately 2% of the total health service budget, equating to nearly £2 billion annually [5]. Importantly, around 70% of these costs arise from unplanned hospital admissions, which are often prolonged, associated with functional decline, and place considerable strain on healthcare resources [5,6].

Reducing avoidable hospitalisations has therefore become a central NHS policy priority, with national strategies emphasising earlier diagnosis, optimisation of guideline-directed therapy, and expansion of community-based care pathways [5,6]. The economic and service burden underscores the need for innovative approaches to managing heart failure in real-world practice, including digital monitoring, integrated multidisciplinary care, and strategies that address persistent health inequalities [4,9].

Aetiology and pathophysiology

CHD remains the leading cause of heart failure in the UK. Progressive narrowing of the coronary arteries due to atherosclerosis reduces myocardial perfusion and frequently culminates in myocardial infarction. The resultant necrosis and loss of viable myocardium trigger adverse left ventricular remodelling, progressive systolic dysfunction, and eventual clinical heart failure [7].

Other important causes include long-standing hypertension, valvular heart disease, arrhythmias such as atrial fibrillation, and both genetic and acquired cardiomyopathies [7]. Risk factors such as diabetes, obesity, smoking, and sedentary lifestyle significantly increase susceptibility to these conditions and accelerate disease progression [9,10].

The pathophysiology of heart failure with reduced ejection fraction (HFrEF) is characterised by structural and neurohormonal maladaptation. Ventricular dilatation and wall thinning impair contractility, while neurohormonal activation particularly of the renin-angiotensin-aldosterone system (RAAS) and sympathetic nervous system initially preserves cardiac output but, with chronic stimulation it promotes sodium and water retention, vasoconstriction, myocardial hypertrophy, and fibrosis [7]. These maladaptive processes elevate filling pressures, leading to pulmonary congestion, peripheral oedema, and multi-organ dysfunction. Although natriuretic peptides are upregulated in response to volume overload, their counter-regulatory effects are insufficient to overcome persistent RAAS and sympathetic overactivity [7].

Guideline-directed medical therapy (GDMT)

Over the past two decades, pharmacological therapy has dramatically improved outcomes in patients with HFrEF. Current European Society of Cardiology (ESC) and National Institute for Health and Care Excellence (NICE) guidelines recommend a “four-pillar” approach that combines ACEIs or ARNIs, beta-blockers, MRAs, and SGLT2 inhibitors [7]. The key heart failure clinical trials are shown in Table 1.

**Table 1:** Key Heart Failure Trials CV: cardiovascular, HF: heart failure, ACEI: angiotensin-converting-enzyme inhibitor, ARNI: angiotensin receptor-neprilysin inhibitor, MRA: mineralocorticoid receptor antagonist, SGLT2i: sodium-glucose cotransporter 2 inhibitors, GDMT: guideline-directed medical therapy

Trial	Intervention	Main Outcome	Year
CONSENSUS [8]	Enalapril (ACEI)	40% mortality reduction in severe HF	1987
PARADIGM-HF [9]	Sacubitril/Valsartan (ARNI)	20% decreased CV death, 21% decreased HF hospitalisation vs enalapril	2014
RALES [10]	Spironolactone (MRA)	30% decreased mortality in severe HF	1999
SHIFT [11]	Ivabradine	Decreased CV death/HF hospitalisation in sinus rhythm	2010
DAPA-HF [13]	Dapagliflozin (SGLT2i)	26% decreased CV death or HF worsening	2019
EMPEROR-Reduced [12]	Empagliflozin (SGLT2i)	25% decreased CV death or HF hospitalisation	2020
STRONG-HF [16]	Rapid GDMT Optimisation	Improved survival with early, intensive up-titration	2022

ACE Inhibitors and ARNIs

ACE inhibitors were first shown to improve survival in severe heart failure in the CONSENSUS trial, which demonstrated a 40% reduction in mortality with enalapril compared to placebo [8]. They have since remained the cornerstone of therapy, reducing both morbidity and mortality in HFrEF [7]. More recently, ARNIs such as sacubitril/valsartan have demonstrated superior efficacy. The PARADIGM-HF trial showed that sacubitril/valsartan reduced the risk of cardiovascular death by 20% and heart failure hospitalisation by 21% compared with enalapril, establishing mortality benefit beyond ACE inhibitors [9].

Beta-Blockers

By attenuating sympathetic nervous system overactivity, beta-blockers improve ventricular function, reduce arrhythmic risk, and decrease mortality by approximately 30-35% [7]. Their role is particularly important in stabilising cardiac remodelling and preventing sudden cardiac death [7].

Mineralocorticoid Receptor Antagonists

MRAs such as spironolactone and eplerenone provide an additional mortality benefit, as demonstrated in the RALES trial, where spironolactone reduced the risk of death by 30% in patients with severe heart failure [10]. Their mechanism involves inhibition of aldosterone-mediated sodium retention, myocardial fibrosis, and adverse ventricular remodelling [7].

Ivabradine

In selected patients in sinus rhythm with elevated heart rates despite beta-blocker therapy, ivabradine has been shown to reduce hospitalisation and improve outcomes. The SHIFT trial demonstrated that ivabradine significantly reduced the composite endpoint of cardiovascular death or hospitalisation for worsening heart failure [11].

SGLT2 Inhibitors

Initially developed for glycaemic control in diabetes, SGLT2 inhibitors such as dapagliflozin have shown substantial benefit in HFrEF irrespective of diabetes status. The DAPA-HF trial demonstrated a 26% relative risk reduction in the combined endpoint of cardiovascular death or worsening heart failure [13], while the EMPEROR-Reduced trial confirmed similar benefits with empagliflozin, showing a 25% relative risk reduction in the composite endpoint of cardiovascular death or hospitalisation for heart failure [12]. These findings establish SGLT2 inhibitors as a fundamental component of GDMT.

Therapy Optimisation

Despite the strength of evidence, underuse and under-titration remain persistent challenges in routine clinical care. Many patients do not receive all four classes at target doses, often due to comorbidities, polypharmacy, or healthcare system constraints. The STRONG-HF trial provided compelling evidence that rapid initiation and up-titration of quadruple therapy, supported by close follow-up and monitoring, is both safe and effective. Patients treated under this intensive optimisation protocol experienced significant improvements in outcomes compared with standard care [10]. However, implementation in routine NHS practice is limited by workforce capacity, infrastructure, and the challenges of scaling such intensive follow-up across diverse patient populations.

Device-based therapy

For selected patients with HFrEF, device-based therapies provide substantial prognostic and symptomatic benefits. Two main modalities are recommended within current ESC and NICE guidelines: ICDs and CRT [7,14].

Implantable Cardioverter-Defibrillators

ICDs are indicated in patients with severe left ventricular systolic dysfunction who remain at high risk of sudden cardiac death despite optimal medical therapy. By detecting and terminating malignant ventricular arrhythmias, ICDs reduce all-cause mortality in appropriately selected patients. Their benefit is most pronounced in those with a life expectancy sufficient to outweigh procedural risks and competing comorbidities [7].

Cardiac Resynchronisation Therapy

CRT is particularly beneficial in patients with symptomatic HFrEF who demonstrate ventricular dyssynchrony, typically defined by a widened QRS duration with left bundle branch block morphology. By restoring coordinated contraction between the left and right ventricles, CRT improves cardiac efficiency, reduces mitral regurgitation, promotes reverse remodelling, and lowers mortality [7]. Patients often experience marked improvement in exercise tolerance and quality of life.

Implementation Challenges

Despite robust evidence, real-world uptake of device therapy remains lower than guideline recommendations. Older adults, those with multimorbidity, and individuals from socioeconomically deprived backgrounds are less likely to be offered ICDs or CRT [7]. Barriers include concerns regarding procedural risk, limited specialist availability, competing mortality risks, and in some cases, patient preference. Addressing these disparities requires improved referral pathways, equitable access to specialist centres, and shared decision-making that balances potential benefits with patient goals of care.

Lifestyle modification and self-management

Lifestyle modification represents a cornerstone of holistic heart failure care, complementing pharmacological and device-based interventions. Both ESC and NICE guidelines emphasise that education and empowerment are critical for enabling patients to actively manage their condition, reduce the risk of decompensation, and maintain functional independence [5,7].

Dietary Measures

Sodium restriction remains widely recommended, with guidance suggesting a target of less than 5 g/day, although evidence for strict thresholds remains mixed. Excessive restriction may impair appetite and quality of life, particularly in frail or elderly patients, and therefore individualised advice is advocated. Fluid restriction, typically to 1.5-2 litres per day in symptomatic patients, can help limit fluid overload and hospitalisation risk [5,7].

Weight Monitoring

Daily weight monitoring is an effective, low-cost strategy for detecting early signs of fluid retention. An increase of 2-3 kg over a few days often precedes clinical deterioration, allowing for timely adjustment of diuretic therapy. NICE and ESC guidelines both recommend this practice as part of routine self-management [5,7].

Exercise and Physical Activity

Structured exercise programmes, such as cardiac rehabilitation, are safe and improve exercise tolerance, quality of life, and psychological well-being. Even in patients with significant limitation, low-to-moderate intensity physical activity is encouraged, with physiotherapist-led programmes tailored to individual capacity. Regular movement also helps counteract the risks of frailty, sarcopenia, and social isolation [7,15].

Smoking, Alcohol, and Comorbidity Control

Smoking cessation, moderation of alcohol intake, and optimisation of comorbid conditions such as diabetes, obesity, and hypertension are vital components of lifestyle management. Each of these factors influences prognosis, and their modification can reduce the risk of recurrent hospitalisation and disease progression [7,15].

Patient Education and Self-Management

Education is central to enabling patients to take ownership of their condition. This includes understanding the purpose of medications, recognising early symptoms of decompensation (such as weight gain or worsening breathlessness), and knowing when to seek medical review. Involving family members or carers is strongly recommended, as this enhances adherence, ensures continuity, and provides additional safeguards in recognising deterioration [5,7].

Evidence and Practice Gaps

While lifestyle measures are widely endorsed, the strength of evidence varies. For instance, sodium restriction lacks strong trial data, and recommendations are increasingly shifting towards pragmatic, patient-centred advice rather than rigid rules. Similarly, while exercise programmes improve outcomes, uptake remains suboptimal due to resource limitations and variable access across NHS services [16]. Ensuring equitable provision of lifestyle support is therefore a key challenge for future practice.

Multidisciplinary team (MDT) approach

**Figure 1:**
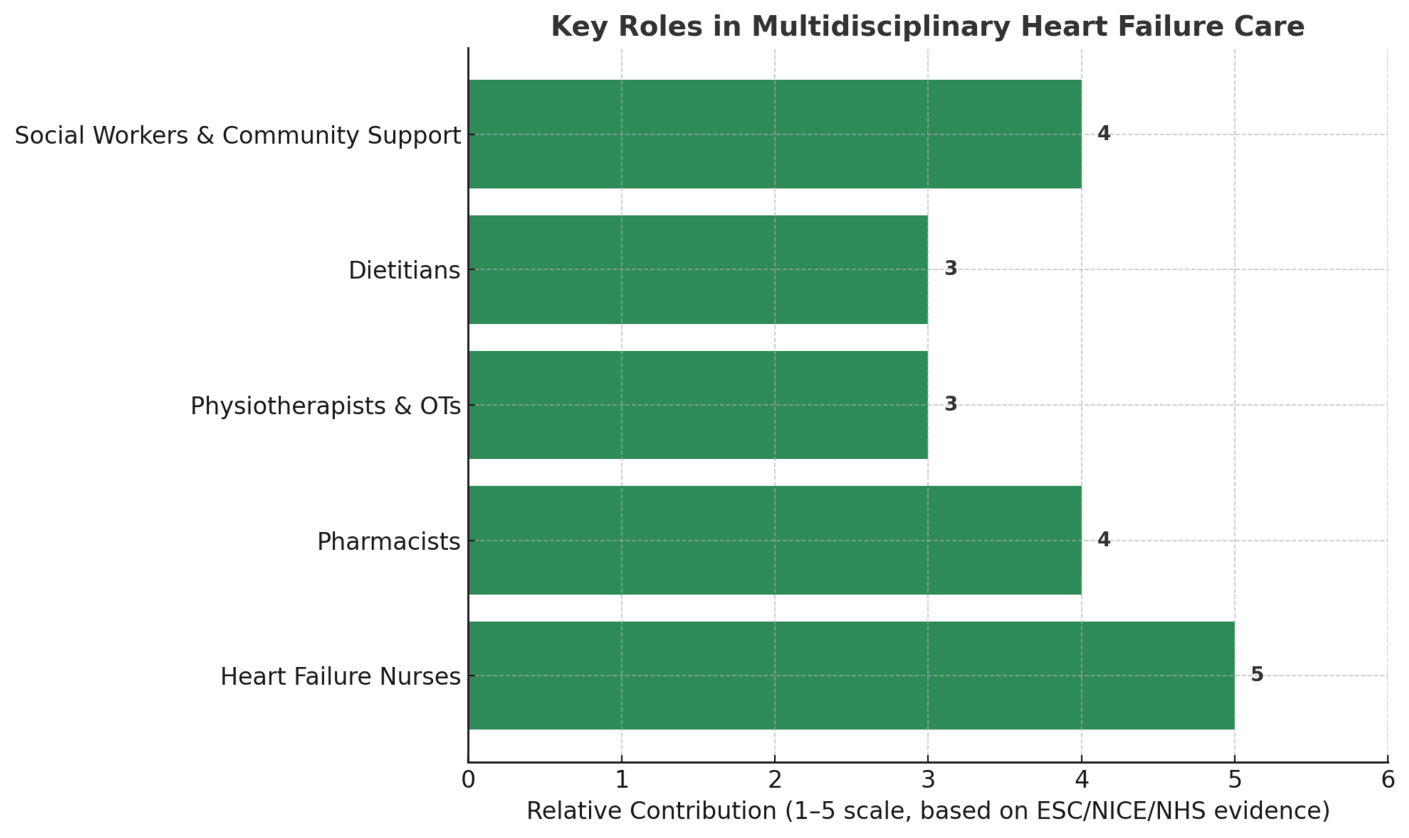
Key Roles in Multidisciplinary Heart Failure Care Evidence Base: European Society of Cardiology (ESC) Guidelines (2021) [7], National Institute for Health and Care Excellence (NICE) Chronic Heart Failure Guidance (2023) [5], and NHS Long Term Plan (2019) [4]. Heart Failure Nurses (score 5) → Audits show they have the strongest role in reducing admissions and optimising therapy. Pharmacists (score 4) → Strong role in managing polypharmacy and adherence. Physiotherapists and Occupational Therapists (score 3) → Proven benefit in functional capacity, but evidence is less robust than nurses/pharmacists. Dietitians (score 3) → Important in malnutrition/obesity management, but impact evidence is moderate. Social Workers and Community Support (score 4) → Critical in tackling inequalities and access issues, strong evidence from NHS service audits. The numbers (1–5 scale) are relative contribution scores derived from strength-of-evidence grading across the above sources (not raw patient counts).

The management of heart failure is inherently complex, requiring expertise from multiple healthcare professionals (Figure 1). No single intervention alone can address the clinical, functional, and psychosocial challenges faced by these patients. Both ESC and NICE guidelines emphasise the value of an integrated MDT in reducing hospitalisations, improving survival, and enhancing quality of life [7].

Specialist Heart Failure Nurses

Heart failure nurses are at the core of MDT-based care. They provide patient education, optimise medication titration, and act as a vital link between hospital and community services. Evidence from national audits demonstrates that patients managed under nurse-led services have lower readmission rates and better adherence to guideline-directed medical therapy (GDMT) [2,7].

Pharmacists

Pharmacists play a crucial role in managing polypharmacy, minimising drug-drug interactions, and simplifying regimens to improve adherence. Their input is particularly valuable in elderly patients with multimorbidity, where inappropriate prescribing and treatment complexity can undermine outcomes [7].

Physiotherapists and Occupational Therapists

These professionals support functional recovery and independence. Physiotherapists deliver safe, tailored exercise rehabilitation, while occupational therapists provide adaptations and strategies that allow patients to maintain activities of daily living. This is particularly important for patients with frailty or reduced mobility [7].

Dietitians

Nutritional optimisation is essential in heart failure, especially for those with comorbid diabetes, obesity, or cachexia. Dietitians provide targeted advice on sodium and fluid intake, weight management, and maintaining adequate caloric and protein intake to prevent malnutrition [7].

Social Workers and Community Support

Heart failure is strongly influenced by social determinants of health, including housing, income, and transport access. Social workers help patients navigate welfare support, arrange home adaptations, and connect them with community resources. Their role ensures that medical care is reinforced by addressing practical and social barriers [7,9].

MDT Outcomes and NHS Strategy

Multiple studies confirm that MDT-led management improves clinical outcomes and is cost-effective, reducing hospital admissions and length of stay [7]. The NHS Long Term Plan strongly advocates for the expansion of community-based MDTs, supported by virtual wards and remote monitoring technologies [4,9]. These models provide continuity of care between clinic visits, enable early detection of deterioration, and align with national priorities to deliver more care in the community.

Barriers to MDT Integration

Despite proven benefits, challenges remain in implementing MDT care consistently. Workforce shortages, variation in service availability, and inequitable access across regions limit its reach. Furthermore, digital health initiatives such as remote monitoring risk widening health inequalities if disparities in technology access and digital literacy are not addressed [13]. Overcoming these barriers requires sustained investment, targeted commissioning, and strategies to ensure equitable access.

Health inequalities

**Figure 2:**
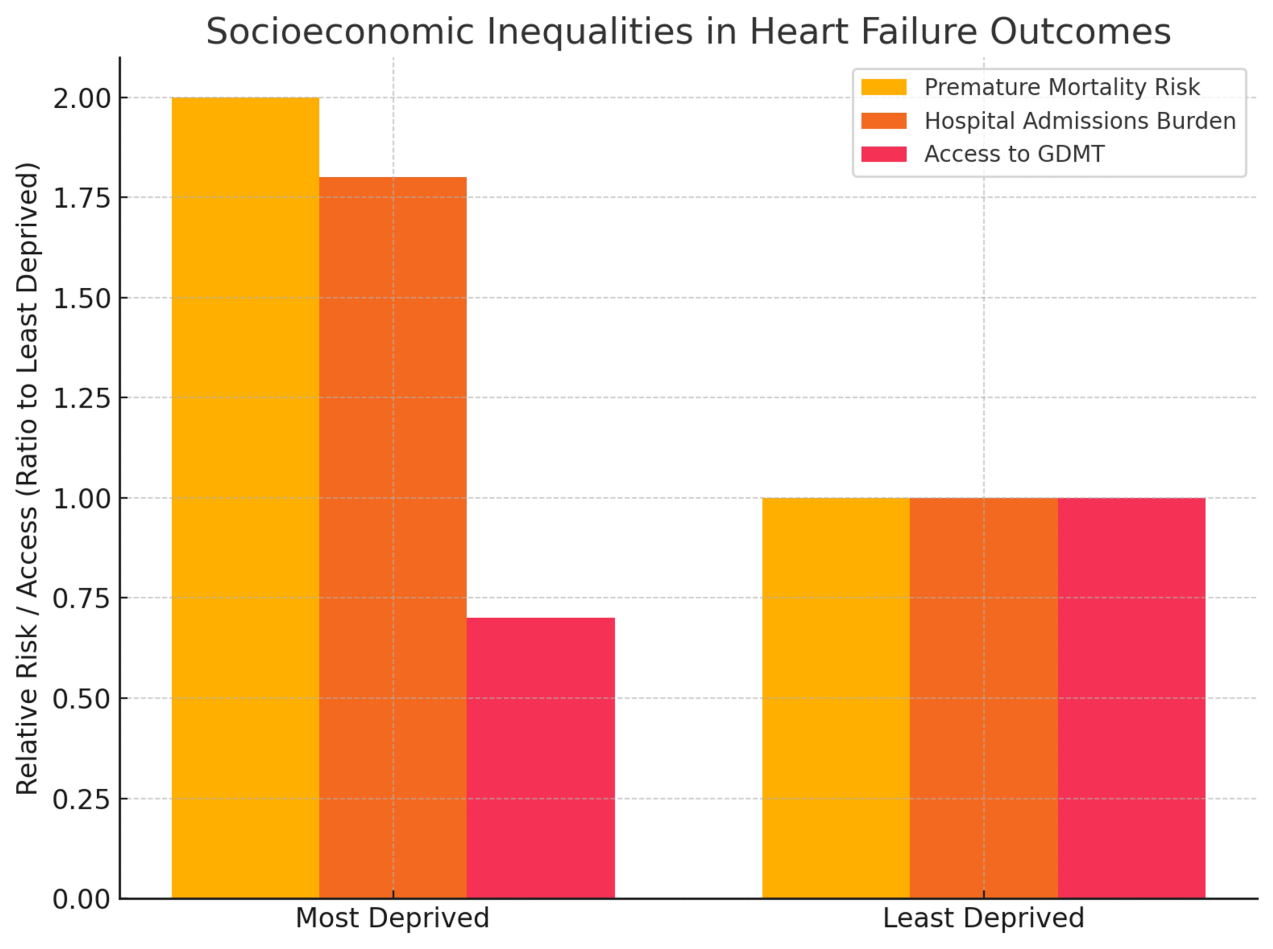
The graph highlights how socioeconomic status directly affects survival, hospital burden, and treatment equity in heart failure. Data Source: Public Health England (CVD profiles, 2021) [14] and NHS reports [4] Mortality Risk: Patients in the most deprived areas are nearly 2× more likely to die prematurely from cardiovascular disease compared to those in the least deprived. Hospital Admissions Burden: Unplanned hospitalisations for heart failure are about 1.8× higher in deprived regions. Access to guideline-directed medical therapy (GDMT): Patients from deprived groups have ~30% lower access to full GDMT, reflecting inequalities in specialist referral and treatment optimisation.

**Figure 3:**
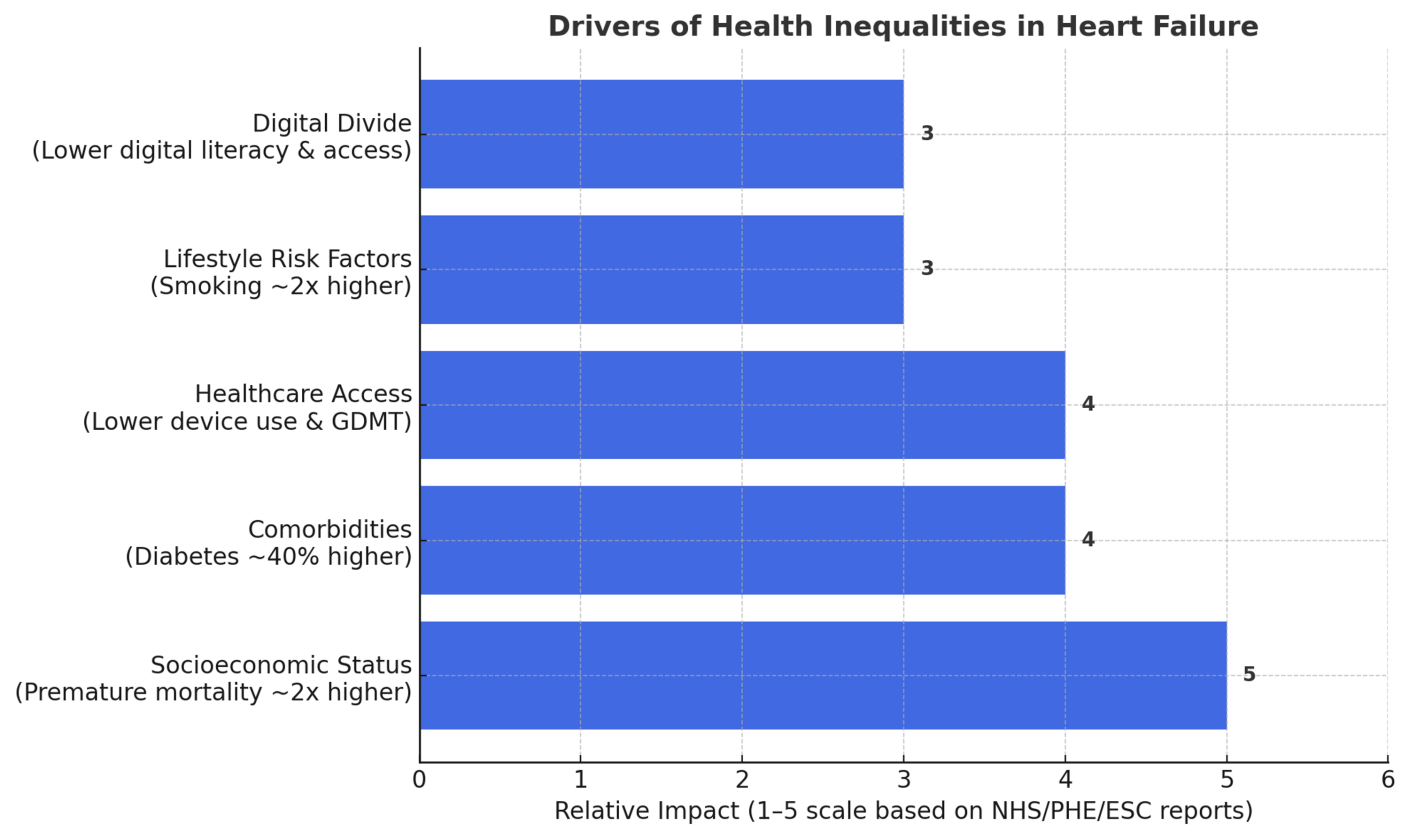
Drivers of Health Inequalities in Heart Failure Socioeconomic Status (Impact = 5): Premature cardiovascular disease (CVD) mortality nearly 2x higher in the most deprived vs least deprived groups (Public Health England (PHE) 2021 [14]; NHS Long Term Plan 2019 [4]). Comorbidities (Impact = 4): Diabetes prevalence ~40% higher in deprived groups, worsening heart failure outcomes (European Society of Cardiology (ESC) Guidelines 2021 [7]). Healthcare Access (Impact = 4): Lower use of guideline-directed medical therapy (GDMT) and device therapies (implantable cardioverter defibrillators/cardiac resynchronization therapy) despite eligibility (NHS Digital 2024 [1]). Lifestyle Risk Factors (Impact = 3): Smoking ~2x more common in deprived groups; obesity and hypertension cluster disproportionately (British Heart Foundation (BHF) 2025 [6]). Digital Divide (Impact = 3): Lower access to remote monitoring/digital health due to reduced device ownership and digital literacy (NHS England 2019 [4]).

Heart failure does not affect all groups equally. The burden of disease, access to care, and long-term outcomes are strongly influenced by socioeconomic status, comorbidities, and wider social determinants of health (Figures 2, 3). Addressing these inequalities has been recognised as a major priority in both national cardiovascular strategies and the NHS Long Term Plan [4,14].

Socioeconomic Disparities

Data from Public Health England show that individuals living in the most deprived areas of the UK are more likely to develop heart failure at a younger age, present with more severe disease, and have poorer survival than those in more affluent areas [14]. These disparities reflect a higher prevalence of risk factors such as smoking, obesity, diabetes, and hypertension, as well as differences in timely access to preventive and specialist services.

Access to Care

Inequalities extend to healthcare utilisation. Patients in deprived regions are less likely to receive guideline-directed medical therapy, less likely to be referred for device implantation, and often experience delays in diagnosis due to limited access to echocardiography and cardiology expertise [14,16]. These gaps highlight the importance of targeted interventions to improve equity in service delivery.

Role of Social Determinants

Beyond medical care, broader determinants such as housing quality, employment, education, and income significantly shape health outcomes. Poor housing conditions can exacerbate respiratory symptoms, while limited income may restrict the ability to maintain a heart-healthy diet or afford transport to appointments. These factors often contribute to recurrent hospitalisation and functional decline despite optimal drug therapy [14].

Digital Divide

The increasing use of remote monitoring, telemedicine, and virtual wards offers great promise for improving care, but risks widening disparities if digital access and literacy are not addressed. Patients in deprived communities and older adults are less likely to have reliable internet access, digital devices, or the skills needed to use them effectively [14]. Unless tailored support is provided, these innovations may inadvertently increase inequality in outcomes.

Policy and Future Directions

Tackling inequalities requires a multifaceted approach that integrates medical, social, and community-based interventions. Strategies include expanding heart failure services in underserved areas, developing culturally appropriate education programmes, and embedding social care support within MDT pathways. Community screening initiatives, particularly targeting high-risk populations, may also reduce diagnostic delays and improve outcomes [15].

By addressing the social gradient in health, the NHS can reduce premature mortality, improve quality of life for disadvantaged patients, and ensure that advances in heart failure care benefit all segments of society.

## Conclusions

Heart failure remains a complex and evolving challenge in modern healthcare, with substantial clinical, social, and economic implications. Advances in pharmacological and device-based therapies have markedly improved survival and quality of life, yet real-world implementation often falls short due to delayed initiation, under-titration, and unequal access to specialist services. Effective management requires not only evidence-based therapy but also structured multidisciplinary input, lifestyle modification, and active patient engagement.

Equally important is the recognition that outcomes are shaped by wider determinants, including socioeconomic status, comorbidities, and access to care. Patients from deprived backgrounds continue to experience higher rates of morbidity and mortality, highlighting the need for equity-driven strategies. Community-based care models, remote monitoring, and digital health interventions hold promise in bridging these gaps, provided they are implemented in ways that do not widen the digital divide.

Looking ahead, the priorities for health systems such as the NHS are clear: ensure rapid and consistent optimisation of guideline-directed therapies, expand access to multidisciplinary services, and integrate social and digital innovations to support patients beyond the hospital setting. By aligning clinical excellence with equitable service delivery, the burden of heart failure can be reduced and long-term outcomes meaningfully improved for patients across diverse populations.
